# Probing the link between cortical inhibitory and excitatory processes and muscle fascicle dynamics

**DOI:** 10.1038/s41598-023-31825-z

**Published:** 2023-03-20

**Authors:** Benedikt Lauber, Wolfgang Taube

**Affiliations:** Department of Neurosciences and Movement Sciences, University of Fribourg, Fribourg, Switzerland

**Keywords:** Motor control, Motor cortex, Neurophysiology

## Abstract

During movements, neural signals are translated into muscle fibre shortening, lengthening or they remain isometric. This study investigated cortical excitatory and inhibitory processes in relation to muscle fascicle dynamics during fixed-end rapid contractions. Fourteen adults performed submaximal and maximal ankle dorsiflexions. Single and paired pulse transcranial magnetic stimulation over the cortical representation projecting to the tibialis anterior (TA) was applied during rest, the activation and deactivation phase of contractions to test for short- (SICI) and long-interval intracortical inhibition (LICI) and intracortical facilitation (ICF). Ultrasound images were taken to measure muscle fascicle dynamics of the superficial (TA_SF_) and deep (TA_DP_) TA compartments. The results show significantly greater maximal shortening velocities (p = 0.003, d = 0.26, CI [4.89, 18.52]) and greater maximal fascicle shortening (p = 0.003, d = 0.86, CI [0.29, 3.13]) in TA_SF_ than TA_DP_ during submaximal dorsiflexions. Significantly lower SICI levels during activation compared to deactivation (p = 0.019, d = 1.12, CI [19.82, 1.76]) and at rest (p < 0.0001) were observed. ICF was significantly greater during activation (p = 0.03) than during rest while LICI did not modulate significantly. Maximal TA_SF_ but not TA_DP_ shortening velocity correlated with SICI levels at activation (p = 0.06) and with the rate of torque development (p = 0.02). The results suggest that SICI might be related to muscle fascicle behavior and that intracortical inhibition and excitation are phase-dependently modulated.

## Introduction

Goal-directed voluntary human movements require refined levels of neural drive generated by the interplay between the activity of cortical excitatory and inhibitory neurons. This neural drive is descending from the motor cortex via the corticospinal pathway to the motoneurons ultimately activating the muscle(s). The most common way to test cortical excitatory and inhibitory input to the muscles is by stimulating the motor cortex with transcranial magnetic stimulation (TMS) and recording the corresponding motor evoked potential (MEP). from the electromyogram (EMG). While on one hand analyzing muscle activity with and without TMS provides valuable insights into neural activation patterns, it neglects the muscle mechanical consequences. Using imaging tools such as ultrasound (US) on the other hand makes it possible to measure muscle fascicle behavior but does not provide detailed insights into the underlying neural principles causing muscle fascicle shortening and lengthening. Thus, a combination of brain stimulation such as TMS and imaging methods like US provides the possibility not only to monitor task- and phase specific changes in excitatory and inhibitory activity but also to relate these neural activation patterns to the behavior of muscle fascicles.

With respect to rapid contractions, previous studies looked at variations in muscle activity measured via surface EMG and related this to muscle fascicle behavior measured via ultrasound. In this sense, Hager et al.^1^ investigated the effect of different ankle joint angles on muscle activation (EMG) and muscle fascicle dynamics (US) during different phases of fixed-end rapid plantarflexions. It has been proposed that the rate of force development (RFD) is initially mainly determined by neural factors such as the speed of motor unit recruitment and the maximal motor unit discharge rates^2^ but that muscle mechanical properties become more important during the later phase of the force–time curve^1^. However, to the best of our knowledge, there exist neither studies investigating rapid contractions by combining TMS and muscle imaging nor any studies describing movement phase dependent modulation in cortical excitation and inhibition during rapid contractions. This is remarkable as rapid contractions constitute a specific skill requiring precisely timed motor commands to rapidly activate and subsequently de-activate the involved muscle(s). Therefore, excitatory and inhibitory processes have to be tuned within very short time periods and these commands need to be translated into adequate muscle fascicle behavior. For slower and repetitive contractions such as during cycling, Sidhu et al.^3^ showed that short-interval intracortical inhibition (SICI) in the leg extensor muscles is low or even absent in the rising phase (activation) of EMG but high in the falling phase (deactivation) of the EMG. For rapid contractions, it is known that when the tibialis anterior (TA) muscle is the agonistic muscle (i.e. dorsiflexion), SICI is almost absent and thus, significantly lower compared to when the TA is the antagonist (plantarflexion) or at rest^4^. The TA muscle is particularly interesting in this context as it was demonstrated that during rapid contractions, the range between cortical excitation and inhibition is much greater compared to other leg muscles such as the soleus^4^. In addition, the TA can be divided into superficial (TA_SF_) and deep (TA_DP_) compartment and it was shown that depending on the kind of electrical stimulation, the two compartments display unique fascicle behavior^5^. More specifically, electrical nerve stimulation resulted in comparable architectural changes of TA_SF_ and TA_DP_ as during voluntary contractions whereas electrical muscle stimulation caused greater changes in the superficial fascicles^5^. In the present study, TMS and US of the TA was used to investigate (a) phase-specific (activation, deactivation) modulations of intracortical facilitation (ICF) as well as short- (SICI) and long-interval intracortical inhibition (LICI) and (b) to test their relation to muscle fascicle behavior of the TA_SF_ and TA_DP_. We hypothesized that SICI and LICI would be low during the activation and high during the deactivation phase while ICF would modulate in the opposite direction. Furthermore, we hypothesized that these modulations where related similarly to the TA_SF_ and TA_DP_. Studying the relationship between cortical inhibitory and faciliatory activity and muscle fascicle behavior is very important because it can provide general insights on the relationship between neural activity and muscle contractile behavior and also because many sports require the performance of rapid contractions.

## Materials and methods

### Study participants

Fourteen subjects (23.2 ± 2.9 years, 8 females, 1.7 ± 0.1 m, 77.5 ± 27.5 kg) agreed to participate in this study. At the beginning of the experiment, subjects were informed and read the information sheet regarding the content of the study and then gave written informed consent. The study was approved by the ethics committee of the University of Freiburg (418/16) and was in accordance with the Declaration of Helsinki.

### Course of the experiment

**Figure 1:**
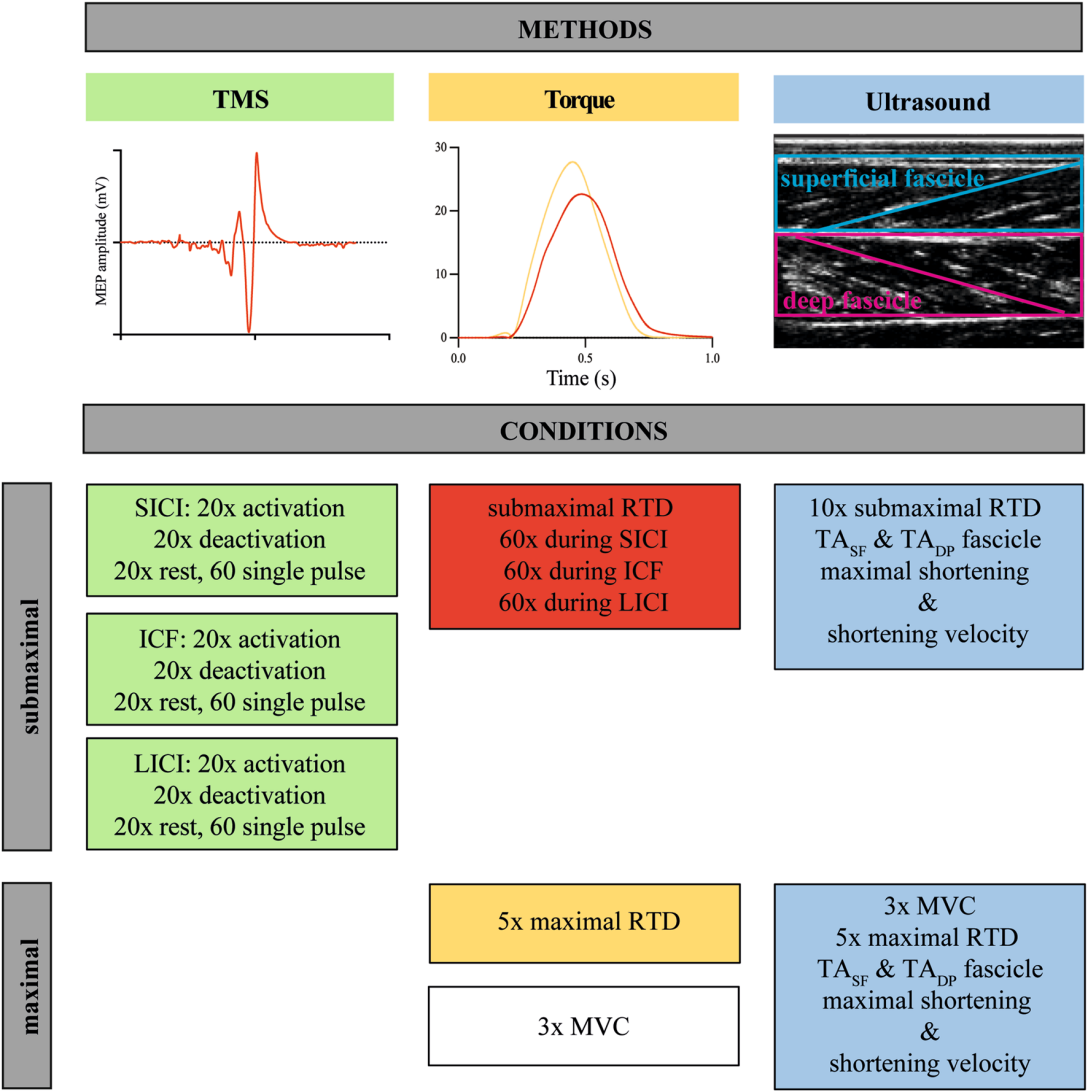
Overview about the methods and experimental conditions of the current study. TMS was measured during rapid submaximal fixed-end dorsiflexions, at rest, and during the activation and deactivation phase of the fixed-end contraction. Ultrasound of the TA_SF_ and TA_DP_ were measured during submaximal as well as maximal rapid contractions and fascicle behavior was analyzed for the activation (shortening) and deactivation (lengthening) phase. Torque was measured during all conditions.

The course of the experiment is summarized in Fig. 1. In brief, each experiment started with the test of the maximal rate of torque development (RTD) followed by the test of the maximal voluntary strength (MVC). This was either followed by the conditions involving transcranial magnetic stimulation (TMS) or the muscle ultrasound (US) measurements. The TMS and the US measurements were performed separately but in the same experimental session.

### Dorsiflexions

For the fixed-end dorsiflexions, subjects were seated in an isokinetic dynamometer (Humac Norm, Computer Sports Medicine Inc. Stoughton, USA) with the hip at 90 degrees and the knee at 20 degrees (almost fully extended). The foot was at an ankle angle of 100 degrees and attached to the footplate of the dynamometer. The foot was strapped to the footplate to exclude movements of the ankle. The hip and the trunk of the subjects were strapped to the backrest of the dynamometer to avoid trunk movements. For familiarization, subjects were allowed to perform a set of 5–7 submaximal rapid contractions. Subsequently, subjects performed 5 contractions and were instructed to contract as fast and as hard as possible and then relax immediately to assess their RTD and the relaxation phase. They were then allowed to rest for 30 s between the contractions (Maffiuletti et al.^10^). Subjects were instructed to initiate the contractions according to the beat of a metronome. Then, subjects performed three MVCs where they were instructed to gradually increase the torque from rest until the maximum over a period of three seconds and then hold the maximal torque for 3 s. There was a rest of 2 min between the MVCs. During the submaximal contractions, subjects were instructed to reach a target line representing 70% of their MVC. The target torque was displayed on a screen as a black line and had to be reached as fast as possible by a red line representing their actually produced torque. During all contractions (RTD, MVC), the actual torque and the target level (for details see procedures) were visually displayed on a computer screen placed 2 m in front of the subjects’ face. Data was sampled at 4 kHz.

### Electromyography (EMG)

After shaving and cleaning the skin with disinfectant, surface EMG was taken from the right m. tibialis anterior (TA), soleus (SOL), medial (MG) and lateral (LG) gastrocnemius using a custom-made EMG system (EISA, University of Freiburg, Freiburg, Germany). According to the SENIAM guidelines, surface electrodes (Blue sensor P, AmbuH, Bad Nauheim, Germany) were attached to the muscles with an interelectrode distance of 2 cm. The reference electrode was placed on the tibial plateau. The EMG recordings were amplified (× 500), bandpass filtered (10–1000 Hz), and sampled at 4 kHz. All electrophysiological as well as torque signals were recorded (AD board: PCIe 7857 Texas instruments 80 MHz) and synchronized using custom made software (Labview, Imago, Pfitec, Freiburg).

### Transcranial magnetic stimulation (TMS)

Transcranial magnetic stimuli over the left hemisphere motor cortex were delivered using a 95 mm focal butterfly-shaped coil (D-B80) attached to a MagPro X100 with MagOption magnetic stimulator (both MagVenture A/S, Farum, Denmark). For all subjects, the initial stimulation point was set approximately 0.5 cm anterior to the vertex and over the midline. The final position of the coil was established by moving the coil anterior and left from the vertex while constantly monitoring the size of the motor evoked potential (posterior-anterior current flow in the motor cortex, with the handle pointing backwards). Once found, the TA motor hotspot position was recorded and constantly controlled by a neuronavigation system (Polaris Spectra, Northern Digital Inc., Waterloo, Canada and Localite TMS Navigator Version 2.0.5, LOCALITE GmbH, Sankt Augustin, Germany).

### Resting and active motor threshold

Resting motor threshold (RMT) was determined by the lowest possible stimulation intensity to evoke MEP peak-to-peak amplitudes greater than 50 μV in three out of five consecutive trials during rest^6,7^. Active motor threshold (AMT) was defined as the stimulation intensity to evoke MEP peak-to-peak amplitudes greater than 100 μV in three out of five consecutive trials^8^ during the fixed-end contractions of the TA. To establish the AMT, a red target line representing 75% of the maximal force was represented on a computer screen in front of the subjects which had to be matched by a black line representing the exerted torque of the subject^4,9^. Subjects were instructed to contract as fast as possible^10^ to reach the target line.

### Procedures

The stimulation was aligned to three different timepoints of the torque-time curve during the submaximal trials. The stimulations occurred (a) during rest, (b) during the ascending phase of the torque signal corresponding to 75% of the maximal torque signal (activation) or (c) in the descending phase of the torque signal again at 75% of the maximal torque signal (deactivation).

Inhibitory and excitatory mechanisms were tested using paired pulse stimulation paradigms where the first pulse acts as a conditioning pulse and the second pulse as the test pulse. The test pulse is then compared to an unconditioned single pulse.

SICI provides information about the activity of (cortical) inhibitory interneurons^11,12^, more specifically GABA-A mediated inhibition^13^. In addition, other processes such as long-interval intracortical inhibition (LICI) potentially reflecting GABA-B inhibitory processes^14^, together with excitatory mechanisms such as intracortical facilitation (ICF), might also contribute to the interplay between excitation and inhibition during the activation and deactivation phase. Therefore, we recorded single and paired pulse TMS responses to assess SICI, LICI as well as ICF together with ultrasound to monitor muscle fascicle dynamics during fixed-end rapid dorsiflexions in the activation and deactivation phase and at rest.

For SICI and ICF, the first TMS-pulse is delivered at intensities below the threshold to evoke a MEP (subthreshold TMS, i.e. 0.7 RMT or AMT) while the second pulse evokes a clearly visible MEP (suprathreshold TMS; i.e. 1.2 RMT or AMT). For LICI, the conditioning as well as the test pulse are suprathreshold pulses (i.e. 1.2 RMT or AMT). Additionally, unconditioned suprathreshold single pulse stimulations were applied during the SICI, LICI and ICF measurements. Depending on the stimulation protocol, the conditioning interstimulus interval was 2 ms for SICI, 100 ms for LICI and 10 ms for ICF. It was ensured that the test pulses always occurred at the same time in the torque-time curve.

#### Procedures during active conditions

After establishing the AMT for the TA for each condition, subjects performed 40 submaximal fixed-end contractions (please see below) during which 20 paired-pulse stimulations (SICI, LICI, ICF) and 20 single-pulse MEPs were applied in a randomized order. This means that for the rest condition, a total of 40 stimulations were applied while in the active conditions, a total of 80 stimulations (40 activation, 40 deactivation) were delivered. Furthermore, the stimulation intensities for SICI and ICF were identical and only the interstimulus interval was different. Importantly, in order to allow comparisons between the results obtained at the different stimulation time points (activation and de-activation phase), the MEP size of the unconditioned pulses (test-pulses) within each stimulation protocol was matched meaning that the MEP size measured during the ascending and descending phase of the torque-time curve was similar. This was important as the background EMG showed – as expected – a significant condition effect between activation and deactivation (p = 0.036).

#### Procedures during rest

Similar to the active conditions, 20 single- and 20 paired-pulse TMS stimulations for each stimulation paradigm (SICI, ICF, LICI) were applied at rest. The only difference was that we used the RMT instead of the AMT. During the stimulations, subjects sat in the isokinetic device in the same position as during the active conditions but were instructed to relax while the EMG was constantly monitored to avoid muscle activation. We included this condition because it allowed us to measure the baseline level of inhibition and excitation.

#### Ultrasound

Images of TA muscle fascicles as well as the superficial, mid- and deep-aponeuroses where obtained via *B-mode* ultrasound (ArtUs,TELEMED, Vilnius, Lithuania) with a central frequency of 8 MHz using a flat ultrasound transducer (LV8-5N60-A2) at a sampling frequency of 138 frames per second and a field of view of 65 mm. Images were collected from the anterolateral aspect of the leg and the probe was fixed to the shank using self-adhesive bandage. US measurements were randomly performed either before or after the TMS protocols to avoid ordering effects and mitigate the unlikely influence of fatigue. The US measurements were synchronized with the torque signal using a 5 V trigger pulse. Ultrasound was recorded during 10 submaximal as well as five maximal RTD trials. Furthermore, US images were also taken during three MVCs.

### Data analyses

#### Strength test

The maximal RTD of the dorsiflexion was defined as the maximal slope of the force torque curve in each trial^15,16^. The best three out of the five trials were averaged and used for comparisons^10^. Maximal voluntary force was defined as the maximal torque signal in each of the three MVCs and the average of the three maxima was calculated.

#### TMS evoked responses

The responses to the TMS stimulation (SICI, LICI, ICF) were quantified by peak-to-peak amplitudes of the conditioned MEPs (paired pulses) in comparison to the unconditioned MEPs (test pulse). SICI and LICI was expressed as percentage inhibition of the conditioned MEP compared to an unconditioned MEP using the formula: 100—(conditioned MEP/unconditioned MEP × 100). ICF was quantified as the percentage facilitation of the conditioned MEP in relation to the unconditioned MEP according to the formula: (conditioned MEP/test MEP × 100) – 100.

#### EMG

Muscle activation was analyzed (root mean square) in a 100 ms window prior to the TMS stimulation and then normalized to the maximal EMG obtained during the MVC measurements.

#### Ultrasound

For the RTD and MVC trials, muscle fascicles of the TA_SF_ and deep TA_DP_ were analyzed using a semi-automated tracking algorithm^17^. Manual adjustments were made where the automatic procedure did not track the fascicle end points well, typically when length changes were large from frame to frame. After the tracking, fascicles length changes were calculated relative to their length during rest. The relative fascicle length was used for comparisons between regions (TA_SF_ vs. TA_DP_) and conditions (submaximal vs. maximal).

#### Relating TMS and US measurements

Because TMS and US measurements were tested in separate sessions in order to ensure optimal measurement conditions for both EMG and US, we compared the RTD between the trials to show that the trials were comparable. Therefore, we calculated the maximal RTD during the TMS from signal onset (2*std of mean signal prior to force onset) until the trigger for the stimulation was released. Then, we used the same time interval for the US trials to calculate the maximal RTD.

### Statistics

The Kolmogorov Smirnov test was calculated to ensure normal data distribution. After that, one-way repeated measures of ANOVA were performed to test for differences in SICI, LICI and ICF at the different movement phases (rest vs. activation vs. deactivation). Due to the activation of the TA during the active conditions in comparison to the rest condition, differences in the unconditioned MEP were tested using a two-way repeated measures of ANOVA with the factors ‘stimulation paradigm’ (SICI, ICF, LICI) and ‘movement phase’ (activation, deactivation). Differences between the unconditioned MEPs during rest (SICI, ICF, LICI) as well as the EMG prior to the stimulation was tested with a one-way repeated measures ANOVA. Excitation/inhibition ratios (ICF/SICI) at the different movement phases were compared by one-way repeated measures ANOVAs. In case of significant differences, Tukey post-hoc tests were calculated. Differences between TA_SF_ and TA_DP_ in maximal fascicle shortening and maximal shortening velocity within the submaximal and maximal trials as well as between submaximal and maximal trials were compared using paired *student t tests* and Cohens *d* was cacluated to estimate effect sizes*.* Additionally, we calculated Pearsons r to identify correlations between the TMS and the US as well as between the US and the torque data during the maximal as well as submaximal trails. As TMS and US trials were recorded in separate tests, we compared the maximal RTD during both trials using *student t-tests.* All data are reported as means ± standard deviation. The level of significance was defined at values p ≤ 0.05 and Prism 9 (GraphPad Software, San Diego, USA) software was used for statistical comparisons.

## Results

### Paired pulse TMS (conditioned MEPs)

**Figure 2:**
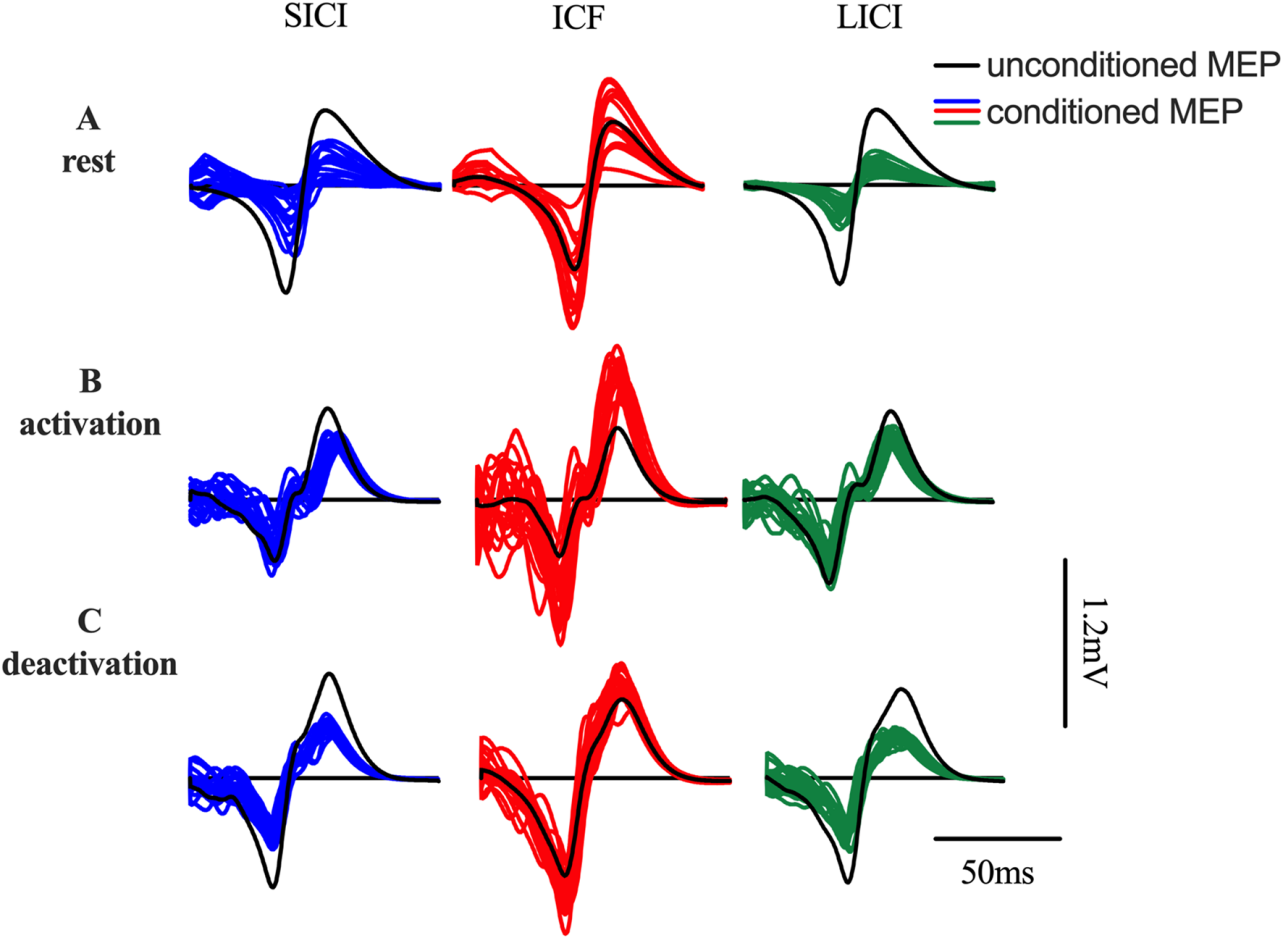
Representative TMS data from a single subject obtained during rest (**A**), activation (**B**) and deactivation (**C**). The colored lines represent individual conditioned (paired-pulse) MEPs for SICI (blue), ICF (red) and LICI (green). The black line shows the mean of the unconditioned (single pulse; test) MEP.

**Figure 3:**
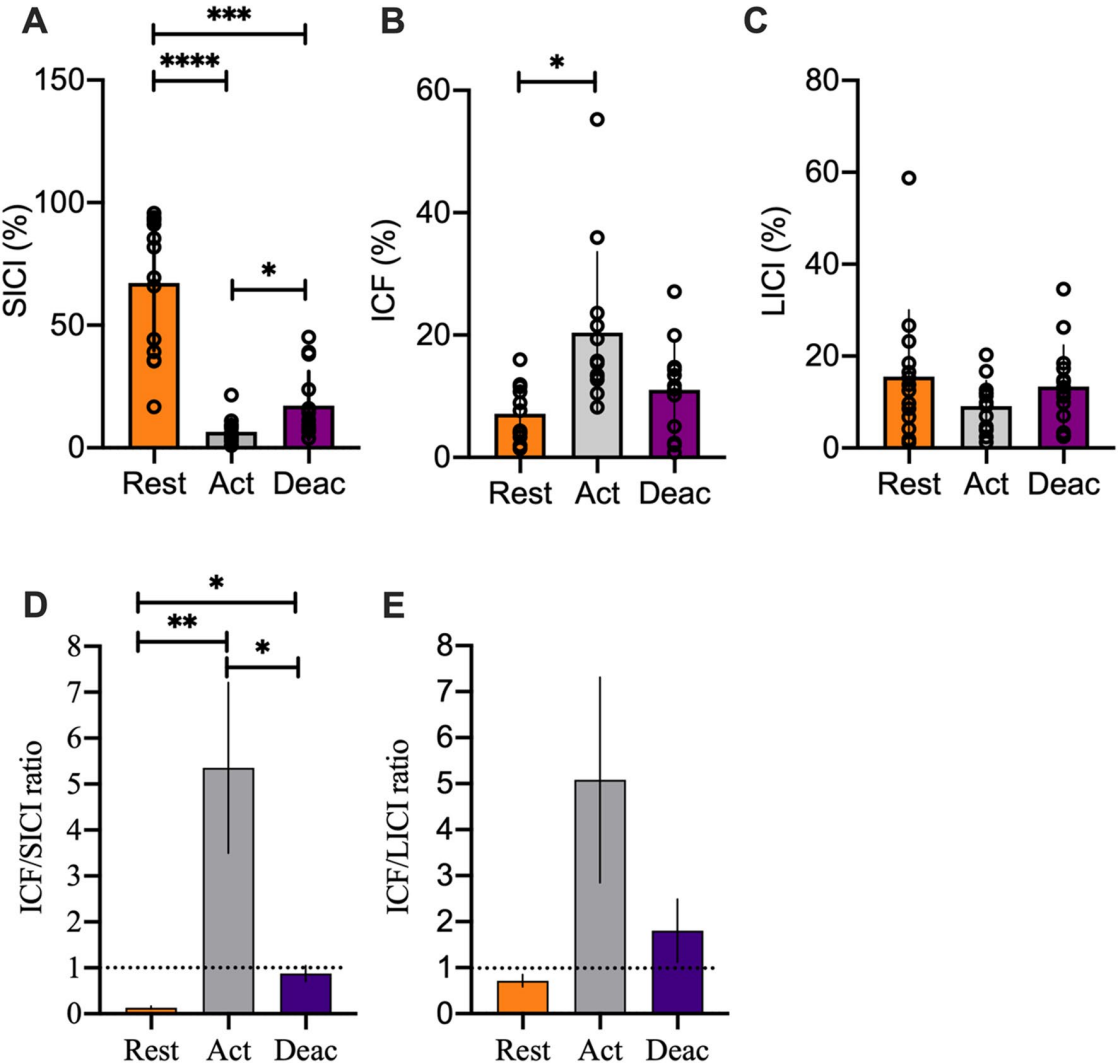
Overview of the group mean TMS responses. The figure shows the group means during rest (orange), activation (grey) and deactivation (purple) for SICI (**A**), ICF (**B**) and LICI (**C**). Panels (**D**) + (**E**) show the ICF to SICI and the ICF to LICI ratio, respectively (****p ≤ 0.0001, ***p ≤ 0.001, **p ≤ 0.01, *p ≤ 0.05).

*SICI:* There was a significant difference in SICI between movement phases (F = 40.38, p < 0.001, R^2^ = 0.76; Fig. 2). Post-hoc tests showed that activation significantly differed from deactivation (p = 0.019, CI − 19.82 to − 1.756). Furthermore, there were also significant differences between rest and activation (p < 0.001, CI 39.20 to 82.25) as well as between rest and deactivation (p = 0.0002, CI 26.65 to 73.23; Fig. 3A).

ICF: There was a significant difference in ICF between conditions (F = 5.33, p = 0.03, R^2^ = 0.33; Fig. 2). Post hoc tests showed that ICF was higher during activation than rest (p = 0.03, CI − 25.59 to − 0.95) but there was no significant difference to deactivation (p = 0.2, CI − 4.69 to 23.39) and between rest and deactivation (p = 0.2, CI − 9.72 to 1.88; Fig. 3B).

*LICI:* There was no significant difference in LICI between the conditions (F = 1.25, p = 0.299, R^2^ = 0.08; Figs. 2, 3C). The results from paired pulse TMS stimulations show a phase-dependent modulation in SICI as well as ICF but not for LICI.

### Excitation/inhibition ratios

In the ICF/SICI ratio, we found a significant condition effect (F = 6.95, p = 0.02). Post-hoc analyses showed that there was a significant greater ratio at activation compared to rest (p = 0.017, CI − 9.34 to − 1.09) and also a greater ratio during deactivation compared to rest (p = 0.001, CI − 1.11 to − 0.36). Finally, the ratio at deactivation was also significantly greater than at activation (p = 0.03, CI 0.45 to 8.50, Fig. 3D). There was no significant condition effect in the ICF/LICI ratio (F = 2.97, p = 0.10; Fig. 3E) but the modulation was very similar to the modulation of the ICF/SICI ratio. This suggest that during rapid contractions of the TA, the balance between inhibition (SICI) and excitation (ICF) plays an important role.

### Unconditioned TMS (test MEPs)

There was no significant interaction effect between the stimulation paradigm and the movement phase in the active conditions (F_2,39_ = 0.7, p = 0.93) highlighting that the control MEP size was comparable. At rest, there was also no statistical difference between the test MEPs in the SICI, ICF and LICI trials (F = 0.93, p = 0.40).

### Ultrasound

#### Submaximal trials

**Figure 4:**
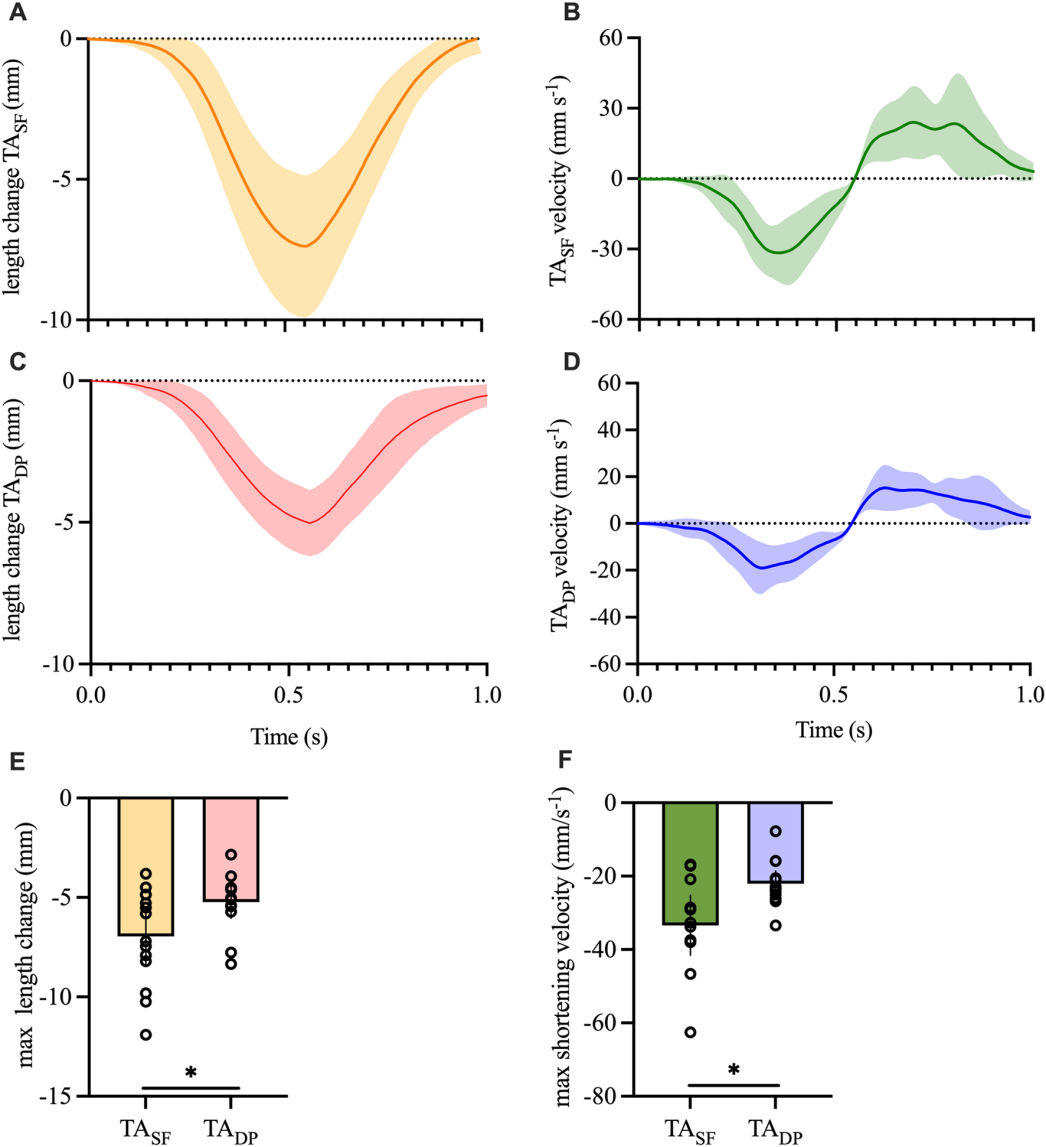
Overview about the measurements obtained with ultrasound for the TA_SF_ and TA_DP_ while measured during submaximal trials. (**A**) + (**C**) show fascicle length changes of the TA_SF_ (**A**) and TA_DP_ (**B**) while (**B**) + (**D**) show the respective shortening velocities. Figure (**E**) shows the average maximal length changes being significantly greater for TA_SF_. (**F**) Average maximal shortening velocities being significantly greater in the TA_SF_ compared to the TA_DP_ (*p ≤ 0.05).

The TA_SF_ shortened to a greater extent than the TA_DP_ (p = 0.02, R^2^ = 0.35, CI 0.29 to 3.13; Fig. 4A–H). The maximal fascicle shortening velocity was also greater in the TA_SF_ than the TA_DP_ (p = 0.003, R^2^ = 0.57, CI 4.88 to 18.52; Fig. 4A–H) suggesting a higher contribution of the TA_SF_ during submaximal rapid fixed-end contractions.

#### Maximal trials

**Figure 5:**
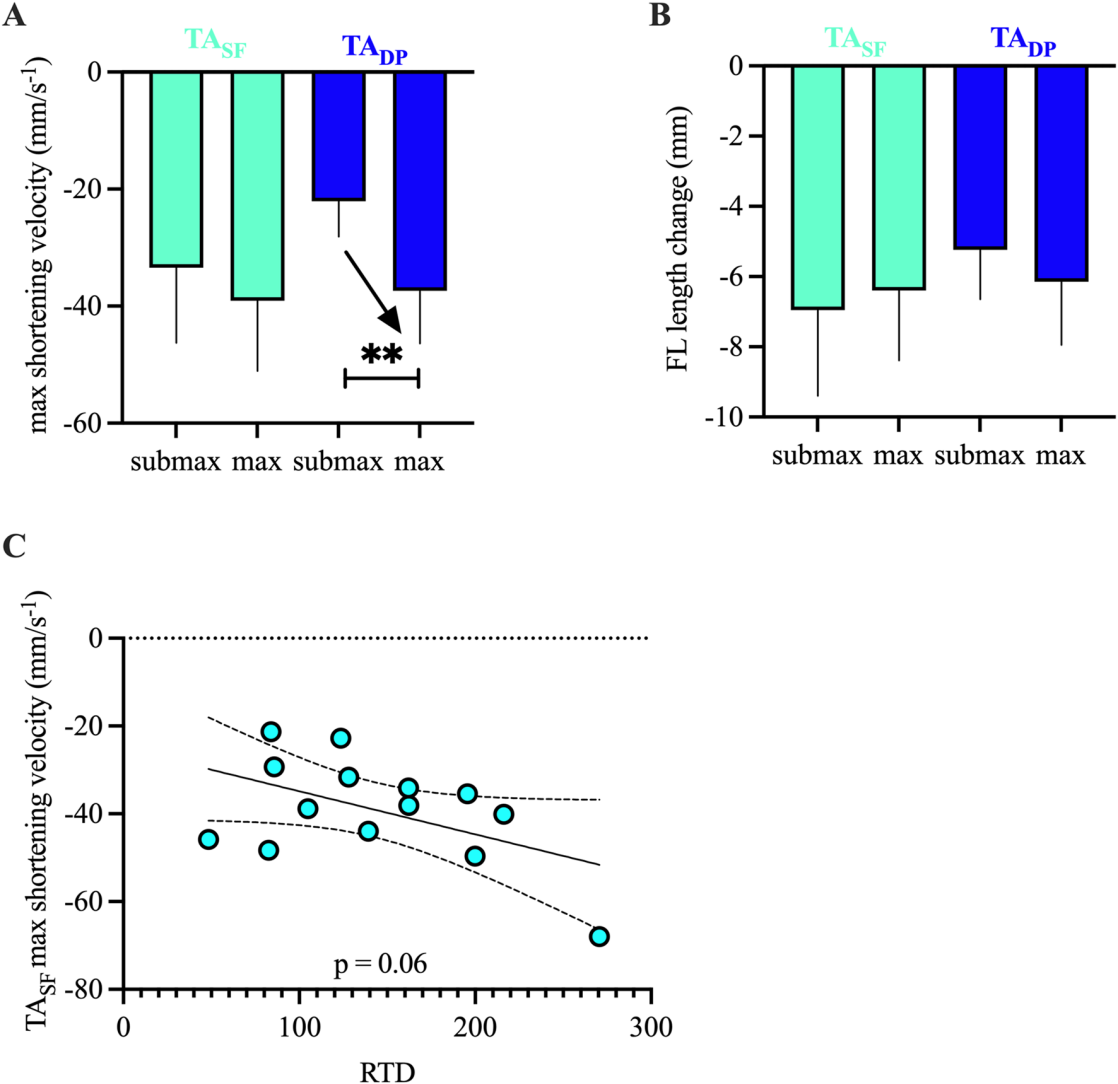
(**A)** displays the maximal shortening velocities of TA_SF_ (cyan) and TA_DP_ (blue) during submaximal and maximal contractions while (**B)** shows the values for the maximal FL length changes. For the TA_DP_ maximal shortening velocity significantly increased from the submaximal to the maximal contractions (**p ≤ 0.01). (**C**) There was trend towards a correlation (p = 0.06) between the TA_SF_ maximal shortening velocity and the maximal RTD (error bars show 95% confidence intervals).

During the maximal trials, there was no longer a significant difference in maximal shortening velocity (p = 0.43, R^2^ = 0.04, CI − 6.41 to 2.93) between the TA_SF_ and the TA_DP_ (Fig. 5A). There was also no difference in the maximal fascicle shortening between TA_SF_ and the TA_DP_ during the maximal trials (p = 0.9, R^2^ = 0.001, CI − 0.10 to 0.11; Fig. 5B). This implies a similar contribution of the TA_SF_ and TA_DP_ during maximal rapid fixed-end contractions.

### Submaximal versus maximal trials

Maximal shortening velocity was significantly greater during the maximal compared to the submaximal trials for the TA_DP_ (p = 0.002, CI 1.14 to 18.93) but not the TA_SF_ (p = 0.99, CI − 13.61 to 12.23; Fig. 5A).

The maximal fascicle shortening did, however, not change from the submaximal to the maximal contractions in the TA_SF_ (p = 0.99, CI − 13.93 to 11.91) as well as the TA_DP_ (p = 0.10, CI − 1.12 to 16.66; Fig. 5B). Furthermore, there was no difference between the shortening and lengthening of the fascicle in the deactivation phase between the submaximal and maximal trials for the TA_SF_ (p = 0.88, CI − 15.71 to 9.12) as well as TA_DP_ (p = 0.83, CI − 12.14 to 6.31).

### Correlations

**Figure 6:**
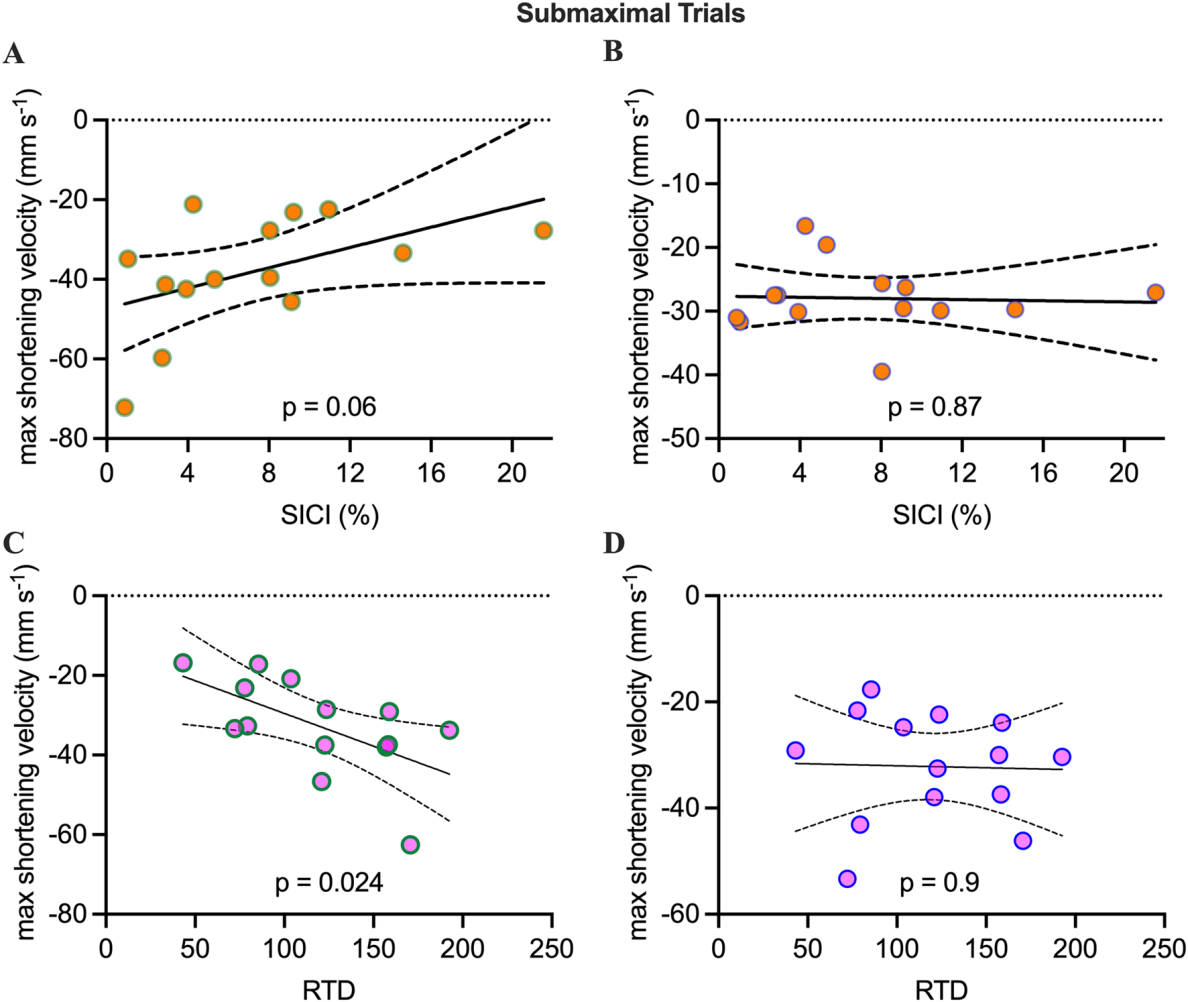
Correlation between the maximal shortening velocity of the TA_SF_ and SICI (**A**) and between maximal shortening velocity of the TA_SF_ and RTD (**C**) obtained during submaximal trials. There were no significant correlations between maximal shortening velocity of the TA_DP_ and SICI (**B**) as well as RTD (**D**). Error bars show 95% confidence intervals.

There was no significant difference between the RTD during the TMS and the US trials (p = 0.62). During the submaximal trials, we found a strong trend for a correlation between SICI in the activation phase and the maximal shortening velocity of the TA_SF_ (r^2^ = 0.25, p = 0.06) but not for the TA_DP_ (r^2^ = 0.002, p = 0.87; Fig. 6A,B). Furthermore, we observed a correlation between the RTD and the maximal shortening velocity of the TA_SF_ (r^2^ = 0.35, p = 0.02) but not between RTD and TA_DP_ (r^2^ = 0.001, p = 0.91; Fig. 6C,D). Even though not significant, the results indicate a relationship between the TA_SF_ shortening velocity and SICI. Furthermore, we show that high levels of RTD require low levels of SICI.

In the maximal RTD trials, there was a trend towards a correlation between the maximal RTD and the maximal shortening velocity of the TA_SF_ (r^2^ = 0.26, p = 0.06) but not between RTD and the TA_DP_ (r^2^ = 0.002, p = 0.61; Fig. 6C).

There were no significant correlations between the fascicle lengthening during the deactivation phase and any TMS parameter.

### Torque

Rate of torque development was significantly greater in the maximal (143.1 ± 61.9 Nm/s) than the submaximal RTD trials (119.1 ± 44.1 Nm/s; p = 0.01).

## Discussion

The aim of the present study was to investigate the relationship between motor cortical excitatory and inhibitory activity and muscle fascicle dynamics during rapid muscular activation and de-activation. The results show a movement phase (activation vs. de-activation) dependent modulation in SICI and ICF and indicates a relationship between low levels of agonistic SICI and high fascicle shortening velocities of the superficial fascicles of the TA. Furthermore, the superficial and the deep compartment of the TA do not behave uniformly. During the submaximal contractions, the maximal shortening velocity as well as the maximal fascicle shortening of TA_SF_ was greater compared to the TA_DP_. When comparing the submaximal with the maximal contractions, only the maximal shortening velocity of the TA_DP_ increased significantly, reaching a comparable maximal shortening velocity than the TA_SF._

### Phase-dependent modulation in excitation and inhibition

During rapid movements such as rapid contractions, it is assumed that the motor cortex needs to provide high levels of descending drive with a high discharge frequency in order to activate a large number of motoneurons ultimately resulting in rapid shortening of muscle fascicles. While there are studies which either exclusively investigated the neural contribution to rapid contractions or solely concentrated on muscle mechanics (for reviews see^10,18^^)^, there is a lack of experiments directly relating neural activation patterns with muscle fascicle behavior during rapid movements.

Most previous experiments investigating neural and/or muscular contribution to RTD focused on the initial part of the torque-time curve (e.g. initial 50 ms) showing that this initial part of the RTD is strongly determined by neural factors such as motor unit discharge rates, recruitment speed, and muscle activation^2,19–21^. In the later phases of RTD, however, the importance of muscle architectural factors like pennation angle^22^ as well as muscle fascicle dynamics^1^ is increasing. Until now, data combining TMS and muscle imaging is scarce and there are studies missing which describe movement phase dependent modulations in cortical excitation and inhibition during rapid contractions. This is the case despite the argument that rapid contractions constitute a highly coordinative skill requiring precisely timed motor commands to rapidly activate and subsequently de-active the involved muscle(s). Therefore, excitatory and inhibitory processes have to be tuned within very short time periods and these commands need to be translated into adequate muscle fascicle behavior. With respect to cortical inhibitory activity during the execution of rapid contractions, Lauber et al.^4^ showed that SICI is much lower during the execution of rapid contractions than at rest and it was suggested that this is important to provide high levels of cortical drive. The present study confirms these findings as SICI is high during rest but almost absent during the activation phase (Fig. 3A). The question still remained, though, to what extend low levels of SICI are actually related to muscle fascicle dynamics and ultimately to the rapid production of dorsiflexor torque. The current results indicate a relationship between SICI, muscle fascicle dynamics and RTD. First, there was a strong indication for a relationship between SICI and the maximal shortening velocity of the TA_SF_ (Fig. 6A) showing that subjects with the lowest SICI level displayed the highest TA_SF_ maximal shortening velocities. This suggests that the ability to reduce cortical inhibition in the activation phase of the contraction is very important for rapid dorsiflexor torque production. While low SICI levels are highly relevant in the activation phase of the movement, a rapid increase in inhibition would be important to rapidly reduce muscle activation when the movement needs to be terminated. This was tested by stimulating in the deactivation phase. As shown in Fig. 3A, SICI is significantly elevated compared to the activation phase. Thus, it seems that SICI can be very rapidly down- (activation phase) or up-regulated (deactivation phase) depending on the phase of the movement. Furthermore, at least for the TA_SF,_ the modulation of SICI is closely related to the ability to rapidly change muscle fiber length during the activation phase.

The high flexibility in up or downregulation of SICI mediated intracortical inhibition is further supported by the finding that during rest, SICI was again significantly higher compared to the other two investigated phases of the movement. Interestingly, neither did LICI modulate very much from rest to activation and between the activation and the deactivation phase of the movement (Fig. 3C) nor did LICI correlate with RTD. While SICI is believed to be a GABA-A mediated inhibitory mechanism^13^, LICI is thought to reflect GABA-B inhibitory processes^14^. Thus, this finding suggests that fast regulations in GABA-A mediated inhibition are more relevant in shaping rapid muscular activation and deactivation than GABA-B mediated inhibition. This finding is well in line with the differential characteristics of GABA-A and GABA-B receptors as SICI was shown to be mainly driven by fast-acting GABA-A receptor types^13,23,24^ while LICI is associated with slower-acting GABA-B receptors^25,26^. In support of this, it was shown that SICI can be modulated within 100 ms during the preparation phase of ballistic dorsiflexions^27^. In needs to be mentioned though, that one study showed potential spinal contributions to LICI in a very limited number of subjects^28^ and we therefore cannot entirely rule out spinal influences to the modulations in LICI shown in Fig. 3C.

As described earlier, rapid contractions are believed to rely on high levels of cortical drive. Therefore, we hypothesized that ICF would play an important role in this process by facilitating motor cortical drive to the TA. The results shown in Fig. 3B partly support this notion as ICF was significantly elevated during the activation phase compared to rest. In the deactivation phase, ICF was lower than in the activation phase, even though it did not reach statistical significance. One explanation could be that in contrast to SICI, ICF modulation is not as rapid and might therefore still be elevated in the early part of the deactivation phase.

In order to quantify the relationship between cortical facilitation (ICF) and inhibition (SICI, LICI) we calculated excitation/inhibition ratios. As shown in Fig. 3D, there is a clear dominance of either SICI or ICF depending on the phase of the movement. While SICI is dominant during rest (ratio 0.13), ICF is dominant when measured during the activation phase (ratio 5.35). During the deactivation phase, SICI and ICF seem to be nearly balanced as the ratio between the two is close to one (ratio 0.87).

### Muscle fascicle dynamics

During the submaximal trials, the maximal shortening as well as the maximal shortening velocity of the TA_SF_ was significantly greater compared to the TA_DP_ compartment (Fig. 4G,H). During the maximal RTD trials, maximal shortening velocity significantly increased only in the TA_DP_ thereby reaching similar levels than the TA_SF_ (Fig. 6A). Maximal shortening also increased from submaximal to maximal in the TA_DP_ while it remained unchanged in the TA_SF_. Thus, it seems that during submaximal trials, the superficial compartment of the TA reaching higher shortening velocities and overall shortening provides a greater contribution to the required torque levels than the deeper compartment. This is supported by the positive correlation between the TA_SF_ maximal shortening velocities and maximal RTD achieved during the submaximal trials (Fig. 4C). When subjects are required to produce maximal dorsiflexor torques, though, the shortening velocity of the TA_DP_ significantly increases reaching similar levels than the ones observed in the TA_SF_ (Fig. 6A). This increase in TA_DP_ shortening velocity might be beneficial to reach maximal RTD levels even though it seems that the TA_SF_ is still the main contributor to maximal RTD as we observed a correlation between RTD and maximal shortening velocities in the maximal trails only for the TA_SF_ (Fig. 5C). Interestingly, maximal shortening velocities of the TA_SF_ only increased slightly from the submaximal to the maximal RTD trials indicating that the TA_SF_ compartment of the TA already works at its optimal shortening velocity at the lower torque levels and is able to maintain this even when a faster rise in torque is required. Interestingly, the maximal lengthening velocities were neither different in the TA_SF_ nor the TA_DP_ in the maximal as well as submaximal trials. This indicates that independent whether subjects are required to perform submaximal or maximal RTD, the fascicle dynamics in the deactivation phase seem to be unaffected. From a functional point of view this makes sense as the participants in the current study were not asked to de-activate differently in maximal and submaximal trials but were instructed to relax (i.e. de-activate) as soon as they had reached the desired toque level. It might, however, be assumed that as soon as rapid foot oscillations are required, the “de-activation” phase is actively supported by the antagonistic muscle and probably results in faster fascicle dynamics in the de-activation phase in maximal compared to submaximal trials.

### Linking excitatory and inhibitory processes with fascicle dynamics

The question remains to what extend are the different excitatory (ICF) and inhibitory (SICI, LICI) processes related to the muscle fascicle dynamics. In order to test this, we correlated the maximal shortening velocities of both compartments of the TA with the parameters obtained from the TMS measurements (ICF, SICI, LICI). For the activation phase, we found a significant correlation between low SICI values and high maximal shortening velocities of the TA_SF_ (Fig. 5A). Furthermore, we also found a correlation between maximal TA_SF_ shortening velocity and the RTD during the submaximal trials (Fig. 6C). This could mean that low levels of SICI allow for high levels of cortical drive as suggested previously^4^ leading to high TA_SF_ shortening velocities which then result in a rapid dorsiflexor torque production. Although TMS and US trials were no recorded simultaneously, the finding that there was no statistically significant difference between the RTD during the submaximal TMS and submaximal US trials strongly suggests that the trials were executed similarly and we therefore assume that the fascicle dynamics were also comparable between the trials. Interestingly, we did not find a correlation between any TMS and US parameters of the TA_DP_. Even though we have no direct evidence, it might be that the differences in fascicle dynamics between the TA_SF_ and the TA_DP_ are related to different activation strategies of the two compartments within the TA. This assumption is supported by a study showing that when subjects were asked to perform dorsiflexions to a similar level than the target torque of submaximal contractions of the present study, the TA_DP_ displayed significantly lower levels of activation compared to the TA_SF_^29^. When subjects were asked to perform maximal RTD contractions, the maximal shortening velocity of the TA_DP_ significantly increased (Fig. 5A) reaching a comparable level than observed in the TA_SF_. Even though speculative, it might be that the neural drive to the TA_DP_ changed to a greater degree than in the TA_SF_ where maximal shortening and maximal shortening velocity remained almost unchanged. Therefore, the more pronounced activation of the TA_DP_ may have been the main driving force for the increased fascicle shortening velocity in the TA_DP_ in the maximal compared to the submaximal trials and might be contribute (amongst other factors such as elevated motor unit firing rates) to the enhanced torque realized during maximal trials.

## Limitations

There are some limitations which should be discussed. During pilot testing, we realized that it was not always possible to perform the TMS and US recordings at the same time as the placement of the electrodes and the placement of the ultrasound probe were in conflict. We therefore decided to perform the measurements separately but in counterbalanced order to avoid any ordering effects and minimize the influence of fatigue. Furthermore, applying TMS during the US recordings would have caused a disruption of the muscle fascicle shortening and it would have not been possible to analyze the muscle fascicle dynamics. It would have also been beneficial to record TMS responses during the maximal RTD trails. However, as maximal contractions cannot be performed very often before fatigue influences the responses but a large number of magnetic stimuli are needed, we did not record any TMS recordings during these maximal trials. Finally, due to the invasive nature, we could not perform intramuscular recordings directly from within the TA_SF_ and TA_DP_. This would have been helpful to sperate the activation profiles of the two compartments during the contractions allowing for a more refined explanation whether differences in recruitment strategies contributed to the different fascicle dynamics seen in the submaximal trials.

## Conclusion

In conclusion, the present study shows movement-phase dependent modulations in cortical excitatory (ICF) and inhibitory processes (SICI, LICI) during rapid fixed-end dorsiflexions. Noteworthy, GABA-A mediated inhibition (SICI) shows the strongest modulations. Furthermore, we show that a reduction in the level of GABA-A mediated cortical inhibition is correlated with the fascicle dynamics of the superficial compartment of the TA. Interestingly, the superficial and deep compartments of the TA seem to be differently activated by the primary motor cortex and seem to be differently influenced by intracortical inhibitory processes. It further appears that the deep compartment is the main driving force when maximizing the contraction velocity as the superficial compartment fascicle dynamics are similar in submaximal and maximal trials.

## Data Availability

For confidentiality reasons, the data is not publicly available but the datasets generated and analyzed during the current study are available from the corresponding author upon reasonable request.
